# The Multiple Faces of Prostaglandin E2 G-Protein Coupled Receptor Signaling during the Dendritic Cell Life Cycle

**DOI:** 10.3390/ijms14046542

**Published:** 2013-03-25

**Authors:** Sandra De Keijzer, Marjolein B. M. Meddens, Ruurd Torensma, Alessandra Cambi

**Affiliations:** 1 Department of Tumor Immunology, Nijmegen Centre for Molecular Life Sciences, Radboud University Nijmegen Medical Centre, Geert Grooteplein 28, Nijmegen 6525GA, The Netherlands; E-Mails: s.dekeijzer@ncmls.ru.nl (S.D.K.); m.meddens@ncmls.ru.nl (M.B.M.M.); r.torensma@ncmls.ru.nl (R.T.); 2 Nanobiophysics, MIRA Institute for Biomedical Technology and Technical Medicine & MESA+ Institute for Nanotechnology, University of Twente, Zuidhorst 164, Dienstweg 1, Enschede 7522ND, The Netherlands

**Keywords:** PGE_2_, G-protein coupled receptors, EP2, EP4, dendritic cell

## Abstract

Many processes regulating immune responses are initiated by G-protein coupled receptors (GPCRs) and report biochemical changes in the microenvironment. Dendritic cells (DCs) are the most potent antigen-presenting cells and crucial for the regulation of innate and adaptive immune responses. The lipid mediator Prostaglandin E2 (PGE2) via four GPCR subtypes (EP1-4) critically regulates DC generation, maturation and migration. The role of PGE_2_ signaling in DC biology was unraveled by the characterization of EP receptor subtype expression in DC progenitor cells and DCs, the identification of the signaling pathways initiated by these GPCR subtypes and the classification of DC responses to PGE_2_ at different stages of differentiation. Here, we review the advances in PGE_2_ signaling in DCs and describe the efforts still to be made to understand the spatio-temporal fine-tuning of PGE_2_ responses by DCs.

## 1. G-Protein-Coupled Receptor Signaling

The ability of cells to communicate with and respond to their external environment is critical for their survival and function. G-protein-coupled receptors (GPCRs) constitute a large and diverse family of proteins, which are crucial intermediates in the transmission and translation of extracellular information into intracellular responses [1]. The GPCR signaling cascade starts by binding a ligand to its receptor, thereby activating downstream signaling pathways, which finally result in complex cellular responses. The signaling and trafficking properties of GPCRs are often highly malleable depending on the cellular context. Receptor-interacting proteins that are differentially expressed in distinct cell types can attribute such plasticity of GPCR function. In addition, the spatiotemporal fine-tuning of a cell’s response to extracellular signals also depends on the probability of interaction between the receptor and its interaction partners, and this is controlled by the organization and lateral mobility of the signaling components within the plasma membrane [2–7]. Mechanisms exist that can control receptor localization and mobility, such as compartmentalization caused by cytoskeletal contacts, lipid environment or protein-protein interactions. Unraveling the mechanisms controlling GPCR signaling may lead to novel therapeutic approaches for treating diseases since GPCRs are amenable drug targets.

### 1.1. Immune Regulation by Dendritic Cells

Immune diseases and cancer are caused by a derailed or ineffective immune system. Understanding the molecular mechanisms that shape an effective immune response is a fundamental question in biology and essential towards the development of novel therapeutics.

Immune responses are orchestrated by a diverse group of functionally specialized, highly differentiated hematopoietic cell lineages. Dendritic cells (DCs) are the most effective antigen (Ag) presenting cells that play a central role in the induction of T-cell-mediated immunity [8]. In addition to activating the immune response, DCs are also decisive in creating tolerance. The pathway that is activated depends on the final balance between incoming signals [9]. In response to antigen uptake and exposure to inflammatory stimuli, DCs undergo a dramatic phenotypic conversion from a tissue resident, Ag-capturing cell to a highly migratory Ag-presenting cell, a process known as DC maturation. Activated DCs run an intricate migration track throughout the body involving the migration from peripheral tissues towards and their entry into the lymphatic vessels, as well as their final positioning in T-cell areas within the lymph nodes where they stimulate naïve T cells and initiate immune responses [8]. DCs are being exploited in the clinic for boosting immunological responses against various cancers by vaccination of cancer patients with *ex vivo*-generated autologous DC loaded with tumor antigens. However, although immunological responses are observed in most studies, clinical responses are limited to a minority of patients. The success of DC-based immunotherapy in inducing cellular immunity against tumors is highly dependent on accurate delivery and trafficking of the Ag-loaded DC to T-cell-rich areas of secondary lymphoid tissues [10,11]. Therefore, in order to improve the clinical outcome, it is of the utmost importance to understand the molecular mechanisms that regulate the differentiation, maturation and migration of DC in order to optimize and steer the immune-regulatory capacities of *ex vivo*-generated DCs.

While extensive research addresses the role of chemokines and cytokines in DC function [12], only recently the role of lipid mediators in DC differentiation and function has been highlighted, when their indispensable role in the DC lifecycle became clear [13–16]. DC differentiation from hematopoietic stem cells (HSCs) and DC function are sensitive to sources of lipid mediators, such as prostaglandins, and DCs express several receptors involved in prostaglandin signaling pathways [17]. Prostaglandin E2 (PGE_2_) is a key modulator of DC differentiation from their specific progenitor cells [18], DC maturation, migration and production of cytokines to influence T cell differentiation [19–22]. Depending on the site of encounter and the maturation stage, PGE_2_ acts both as an immunoactivator and as an immunosuppressor in DCs, exerting a stimulatory function for immature DCs in peripheral tissues [19], and an inhibitory function for mature DCs in the lymph nodes [23]. Finally, PGE_2_ differentially regulates cytokine production by DCs as a response to the context and changes in the microenvironment [22,24,25].

### 1.2. PGE_2_ Signaling

The lipid mediator PGE_2_ is a cyclooxygenase (COX) metabolite of arachidonic acid and exhibits the most versatile actions in a wide variety of tissues, modulating various pathological and physiological activities, such as cancer, fever, inflammation, atherosclerosis, blood pressure, stroke, and reproduction [26,27]. Arachidonic acid is modified by the constitutively active COX-1 and the inducible COX-2 producing prostaglandin H, which is further converted by three different PGE synthases, a cytosolic PGE synthases (cPGES) and two membrane bound PGE synthases (mPGES1 and mPGES2). cPGES and mPGES2 are constitutive active, whereas mPGES1 is inducible [28]. Finally, PGE_2_ is degraded by 15-hydroxyprostaglandin dehydrogenase (15-PGDH), the hydroxyl group is oxidized into 15-keto metabolites which exhibit greatly reduced biological activities [29,30].

PGE_2_ biological actions have been attributed to its interactions with specific GPCRs localized at the plasma membrane [17]. PGE_2_ is believed to act in an autocrine and a paracrine manner via a family of four cell surface or nuclear membrane GPCRs termed EP1–EP4, with distinctive signaling pathways [31]. EP1 is coupled to Gαq/p and activates phosphoinositide-phospholipase C (PLC), which ultimately leads to an increase in intracellular Ca^2+^[32]. EP2 and EP4 are coupled to the stimulatory Gαs, which leads to an increase in intracellular adenosine-3′,5′-cyclic monophosphate (cyclic adenosine monophosphate or cAMP) upon activation. Additionally EP4 has been described to be coupled to a Pertussis toxin-sensitive inhibitory G-protein, Gαi [33]. This will be discussed in more detail in the next paragraph. EP3 exists in three isoforms, all activating Gαi, which decrease cAMP production. Mouse studies suggest that the activation of these receptors induce multiple physiological functions: for example it mediates stress responses [34,35], it facilitates ovulation and fertilization [36], it regulates duodenal secretion [37] and it induces bone formation [38]. The specificity and diversity of PGE_2_ effects can be explained by the local PGE_2_ concentration due to the balance between its COX2-regulated synthesis and 15-PGDH-driven degradation, along with the characteristic expression pattern of different EP receptors with distinctive signaling mechanisms.

PGE_2_ stimulates a broad spectrum of responses throughout most immune cells as reviewed in [39]. For example, PGE_2_ selectively suppresses effector functions of macrophages and neutrophils and the Th1-, CTL-, and NK cell-mediated type 1 immunity at micromolar concentrations and stimulates Th1 and Th17 at nanomolar concentrations [40]. In addition, PGE_2_ supports differentiation, maturation and migration of DCs but suppresses their ability to attract naive, memory, and effector T cells. PGE_2_ regulates the immune response towards Th2 and Th17 immunity and enhances the local accumulation of regulatory T cells and myeloid-derived suppressor cells [41,42].

**Figure 1:**
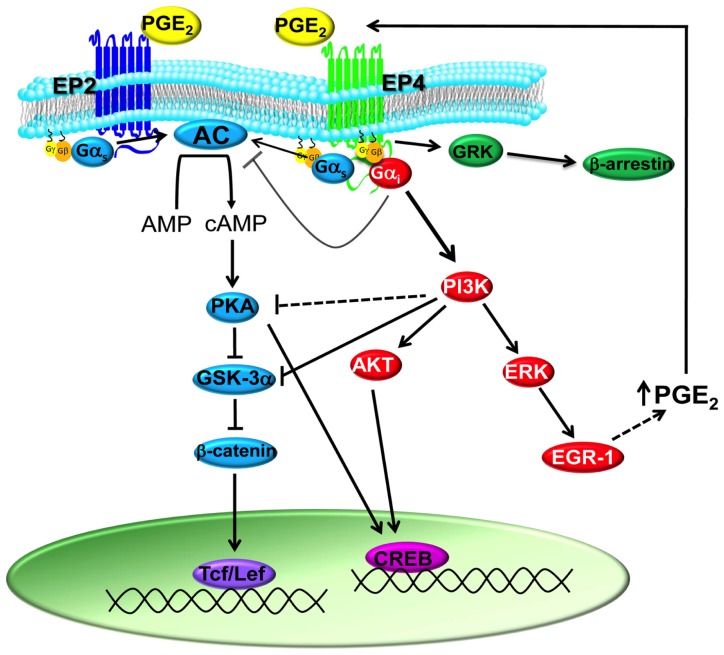
Signaling pathways activated by PGE2 stimulation of the human EP2 and EP4 prostanoid receptors. Phosphorylation of GSK-3α via either PKA or PI3K signaling pathway inhibits the kinase activity of GSK-3α. Inhibition of GSK-3α stabilizes β-catenin that results in a decrease in its degradation and promotes β-catenin nuclear translocation and transcriptional activity of Tcf/Lef-regulated genes. Activation of either PKA or PI3K signaling pathway leads to phosphorylation of the transcription factor CREB regulating cAMP responsive gene expression. Activation of the PI3K/ERK pathway induces functional expression of EGR-1, known to regulate PGE2 synthase. In addition, PI3K signaling pathway inhibits the activity of PKA.

Dependent on DC subtype and species (human or mouse), DCs were shown to express either EP2 and EP4 or all four EP receptors, however PGE_2_ exerts its effects only via EP2 and EP4 (Figure 1) [19,21,26,43,44]. The distinguishing feature of the EP2 and EP4 receptors is that their signaling is predominantly transduced by Gα_s_, through which receptor activation is associated with an increase in adenylate cyclase activity and subsequently elevated intracellular cAMP levels [45,46]. However, EP4 ligation induces a weaker stimulation of intracellular cAMP when compared to the ligation of EP2 expressed at similar levels, although EP4 is known to have a higher affinity for PGE_2_[47]. The production of cAMP and subsequently protein kinase A (PKA) leads to the phosphorylation of glycogen synthase kinase-3 (GSK-3) stimulating Tcf/Lef transcriptional activity. However, although EP4 is able to activate this signaling pathway, EP4 primarily induces Tcf/Lef transcriptional activity via a phosphatidyl-inositol 3 kinase (PI3K)-dependent pathway [48] through activation of Gαi [33,49]. In addition, EP2 and EP4 differentially regulate the PGE_2_-mediated phosphorylation of the cAMP response element–binding protein (CREB), central to the regulation of cAMP responsive gene expression: EP2 stimulates the PKA- and EP4 mainly the PI3K-dependent pathway [50]. In contrast to EP2, EP4 induces the functional expression of early growth response factor-1 (EGR-1) via the PI3K/MAPK signaling pathway [47], which can result in the expression of PGE_2_ synthase [51]. This can act as a positive feedback loop in which ligation of EP4 by PGE_2_ leads to an increase in PGE_2_ production via PGE_2_ synthase. Another distinguishing feature of EP4 is its ligand-induced desensitization and internalization [48,52], depending on elements present in the carboxyl terminus of EP4. The carboxyl terminus of EP4 also contains sites for potential phosphorylation by G-protein coupled receptor kinases (GRK) [53], and arrestin-2 binding promotes EP4 internalization [54]. However, so far mutation of multiple potential GRK phosphorylation sites did not alter agonist-induced internalization [55], suggesting a different or more complex arrestin-EP4 binding mechanism [54]. EP2 has a much shorter C-tail than EP4, which could be a possible explanation for its desensitization- and internalization resistance and lack of arrestin-2 binding. Indeed, an arrestin mutant that binds and desensitizes regardless of phosphorylation status of the receptor did promote EP2 internalization and attenuate EP2 receptor signaling [54].

### 1.3. PGE_2_ and Hematopoiesis

PGE_2_ has a prominent role in controlling the number of hematopoietic stem cells (HSCs) from the bone marrow [56,57], but also HSC present in cord blood [58]. Although all four PGE_2_ receptors were expressed in HSC at protein as well as at mRNA level [59], in the hematopoietic system only EP4 seems operative as recently shown [60]. *In vitro* exposure of HSCs to PGE_2_ leads to better homing, survival and proliferation. The exposure of HSCs has profound influences on the enhanced expression of the C-X-C chemokine receptor type 4 (CXCR4) and survivin while the activity of caspase-3 is down-regulated, both processes inhibit apoptosis [61]. The CXCR4 receptor enhances the migration to stromal cell-derived factor-1 (SDF-1) *in vitro* and homing to the bone marrow *in vivo*[59]. Treatment *in vitro* and *in vivo* of HSC with PGE_2_ results in an increase in stem cell numbers [57]. Dissecting the identity of the responding HSC showed that long term HSCs were unaffected by PGE_2_ and that the increase in HSC number was due to an expansion of the short term HSCs [62]. The latter HSC has less renewal capacity. Presently it is unknown if the long term HSC lacks the receptors for PGE_2_ or whether other downstream processes are modified. PGE_2_ signaling raises the level of β-catenin that is part of the Wnt signaling pathway that drives hematopoiesis [63]. The link between PGE_2_ and Wnt was pinpointed at LGR5, a molecule expressed by (cancer) stem cells and a Wnt target. The level of expression of LGR5 was upregulated by PGE_2_[64]. Recently, it was shown that the upregulation of β-catenin is modulated via EP4 only [60]. Furthermore, PGE_2_ appears to have a dual effect by stimulating the HSC but also the HSC supporting niche, again via the EP4 receptor [60].

After a massive expansion step, HSC differentiate into the eight different blood cell types. Most of these blood cells are insensitive to PGE_2_ since blocking of PGE_2_ production does not influence the differentiation of T cells, B cells and NK cells. In contrast, the number of monocytes increases and the number of DCs decreases after blocking PGE_2_ synthesis with indomethacin [18]. Optimal development of DCs is regulated by PGE_2_ via the EP1 and EP3 receptor. Triggering increases the receptor for Flt3, which is an important cytokine in DC development [60]. Thus, PGE_2_ has regulatory properties on several stages of development of the hematopoietic system, these modulating effects being mediated by different receptor expression patterns.

### 1.4. PGE_2_ Responses in Dendritic Cells

Various studies have demonstrated a multifaceted response of DCs to PGE_2_. In particular, it recently became clear that the timing and extent of DC exposure to this lipid determine different cellular outcomes. *Ex vivo*, DCs can be generated through differentiation of peripheral blood monocytes in the presence of interleukin-4 (IL-4) and granulocyte macrophage colony-stimulating factor (GM-CSF) [65,66]. These moDCs are a well-established system to study DCs and they are currently also exploited in several anti-tumor clinical trials [10,67]. Differentiation of inflammatory monocytes into DCs has been shown to also occur *in vivo*[68], thus making the moDCs a valid model cell system.

The generation of DCs from peripheral blood monocytes has been shown to be inhibited in the presence of PGE_2_, either exogenously added or secreted by co-cultured mesenchymal stem cells [69]. More recently, Kalinski and colleagues demonstrated that a positive feedback loop between PGE_2_ and its synthesizing enzyme COX2 is able to redirect the differentiation of monocyte cultures towards stable myeloid-derived suppressor cells, which have opposing role in the immune system as compared to DCs [70]. In contrast to its inhibitory effects on monocytes, PGE_2_ exhibits an activating function on the immature moDCs. In fact, PGE_2_ is a key regulator of DC maturation, in particular responsible for the acquisition of a migratory phenotype. As a first step towards the transition from an adhesive to a highly migratory state, DCs dissolve specific integrin- and actin-rich adhesive structures called podosomes within minutes after PGE_2_ stimulation [20,71]. This fast response to PGE_2_ is mediated by elevation of cAMP intracellular levels, activation of the small GTPase RhoA and subsequent induction of actomyosin contraction ultimately leading to fast podosome dissolution [21]. Combined with proinflammatory cytokines, PGE_2_ specifically upregulates the surface expression levels of the chemokine receptor CCR7, which is responsible for the chemotactic responsiveness of DCs to lymph node-derived chemokines such as CCL19 and CCL21 [72]. Furthermore, prolonged incubation of DCs with PGE_2_ induces expression of matrix metalloproteinase 9 (MMP-9), which together with CCR7 is responsible for the directional migration of DCs to draining lymph nodes [73,74]. It should be noted that to enable DC chemotaxis, PGE_2_ addition is absolutely required at early time points of maturation, as addition of PGE_2_ during terminal maturation stages is no longer effective [19].

Besides stimulating DC migration, PGE_2_ plays also a role in enhancing the T cell stimulatory capacity of DCs by inducing the upregulation of costimulatory receptors such as OX40L, CD70 and 4-1BBL early during DC maturation that results in an increased capacity to induce proliferation of CD4+ and CD8+ T cells [75], despite the concomitant induction of several suppressive factors like thrombospondin-1 [76] and IDO [77], known to suppress T cell proliferation and survival promoting tolerance. In addition, DC function is influenced by the production of specific cytokines. PGE_2_ induces IL-10 production, a known inhibitor of DC maturation [78] and suppresses the production of IL-12, shifting the balance from a Th1 to a Th2 response [79,80]. A very recent study by Woodward and colleagues demonstrated that PGE_2_ can differentially regulate DC production of cytokines depending on the EP receptor that is stimulated [81]. For example, low concentrations (up to 10 nM) of PGE_2_ appear to stimulate the Th17 response supporting IL-23 release via EP4 [82], whereas at concentrations higher than 50 nM PGE_2_ inhibits IL-23 production in an EP2 dependent manner [81]. These novel findings indicate that subtle changes in PGE_2_ concentration in the extracellular microenvironment can specifically activate one particular receptor thus differentially modulating cellular responses.

Interestingly, the effects of PGE_2_ on naturally occurring DC subsets seem to be quite diverse. For example, signaling of PGE_2_ through EP4 was found to facilitate the initiation of skin immune responses by enhancing maturation and migration of Langerhans cells [43]. In contrast, on human plasmacytoid DCs (pDCs) PGE_2_ has been shown to potently inhibit secretion of IFN-alpha by pDCs upon stimulation with Toll-like receptor ligands, with subsequent decreased secretion of Th1 cytokines by co-cultured T cells [83]. PGE_2_ inhibits IFN-alpha secretion and Th1 costimulation by human pDCs via EP2 and EP4 engagement [84]. PGE_2_ can therefore be considered as a negative regulator on human pDCs [85]. Considering the large variety of properties and functions of the various DC subsets, might lead to multiple cellular responses. This once again highlights the complexity and multifaceted action of PGE_2_ signaling in DCs.

## 2. Outlook

**Figure 2:**
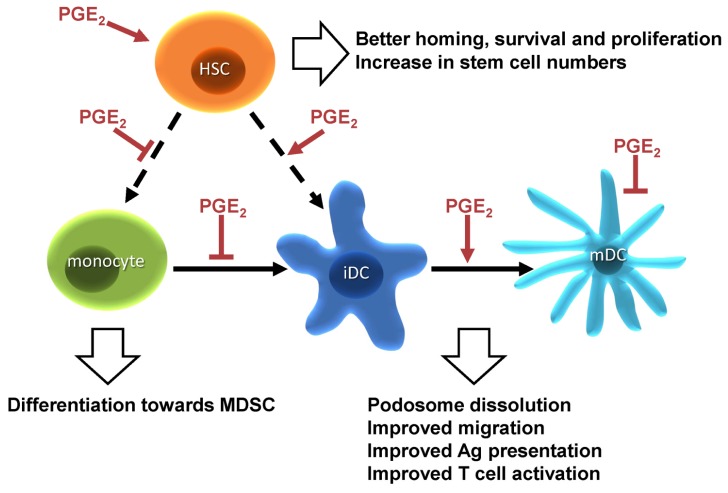
The multifaceted roles of PGE2 during the lifecycle of dendritic cells.

PGE_2_ acts both as an immunosuppressor and an immunoactivator throughout the lifecycle of DCs (Figure 2). Therefore, knowledge on the complexity of PGE_2_ signaling is necessary in order to predict, control and intervene in the PGE_2_ response in experimental and possibly clinical settings. The different cellular effects mediated by PGE_2_ in DCs marginally depend on the expression pattern of the EP receptors. Although differentiation of DCs seems regulated via EP1 and EP3, the function of DCs during their lifecycle is always regulated via EP2 and EP4. Therefore, the expression levels of EP2 and EP4, their different affinity for PGE_2_ and the extent of activation of the different signaling pathways downstream of EP2 and EP4 will enable a DC to tune cellular outputs in response to PGE_2_. The extent of EP2 and EP4 activity is critically dependent on the probability of interaction between the receptor and receptor-interacting proteins, like Gαs, Gαi and arrestin, and subjected to precise regulation of localization in time and space. Furthermore, cross talk of the EP2 and EP4 signaling pathways might occur as well as cross talk by different receptors, influencing the availability of receptor interacting partners or downstream signaling proteins. To fully understand PGE_2_ regulated processes in DCs, all individual molecular interactions should be followed in space and time, which would allow us to build a dynamic and quantitative signaling network connectivity-map for prediction of the DC response to PGE_2_ based on parameters like for example, receptor numbers, affinities *etc.* With the rapid development and improvement of high-resolution bioimaging techniques suited for the investigation of fast signal transduction processes at the molecular level [86], we expect to be able to simultaneously investigate multiple molecular interactions involved in the PGE_2_ receptor signal cascade, which might represent a paradigm for other GPCR signaling pathways.

## Abbreviations

Gβ: Gγ, heterotrimeric guanine nucleotide-binding proteins
Gαs: stimulatory guanine nucleotide binding protein
Gαi: inhibitory guanine nucleotide binding protein
AC: adenylate cyclase
AMP: adenosine monophosphate
cAMP: cyclic 3,5-adenosine monophosphate
PKA: protein kinase A
GSK-3α: glycogen synthase kinase-3
PI3K: phosphatidyl-inositol 3 kinase
Akt: also known as Protein Kinase B
CREB: cAMP response element–binding protein
Tcf: T-cell factor
Lef: lymphoid enhance factor
ERK: extracellular signal-regulated kinase
EGR-1: early growth response protein 1
GRK: G-protein coupled receptor kinase

## References

[1] Pierce K.L., Premont R.T., Lefkowitz R.J. (2002). Seven-Transmembrane receptors. Nat. Rev. Mol. Cell Biol.

[2] Allen J.A., Halverson-Tamboli R.A., Rasenick M.M. (2007). Lipid raft microdomains and neurotransmitter signalling. Nat. Rev. Neurosci.

[3] De Keijzer S., Serge A., van Hemert F., Lommerse P.H.M., Lamers G.E.M., Spaink H.P., Schmidt T., Snaar-Jagalska B.E. (2008). A spatially restricted increase in receptor mobility is involved in directional sensing during Dictyostelium discoideum chemotaxis. J. Cell Sci.

[4] De Keijzer S., Galloway J., Harms G.S., Devreotes P.N., Iglesias P.A. (2011). Disrupting microtubule network immobilizes amoeboid chemotactic receptor in the plasma membrane. Biochim. Biophys. Acta.

[5] Cambi A., Joosten B., Koopman M., de L.F., Beeren I., Torensma R., Fransen J.A., Garcia-Parajo M., van Leeuwen F.N., Figdor C.G. (2006). Organization of the integrin LFA-1 in nanoclusters regulates its activity. Mol. Biol. Cell.

[6] Ganguly S., Pucadyil T.J., Chattopadhyay A. (2008). Actin cytoskeleton-dependent dynamics of the human serotonin1A receptor correlates with receptor signaling. Biophys. J.

[7] Jaqaman K., Kuwata H., Touret N., Collins R., Trimble W.S., Danuser G., Grinstein S. (2011). Cytoskeletal control of CD36 diffusion promotes its receptor and signaling function. Cell.

[8] Banchereau J., Steinman R.M. (1998). Dendritic cells and the control of immunity. Nature.

[9] Steinman R.M. (2003). Some interfaces of dendritic cell biology. APMIS.

[10] Figdor C.G., de Vries I.J., Lesterhuis W.J., Melief C.J. (2004). Dendritic cell immunotherapy: Mapping the way. Nat. Med.

[11] De Vries I.J., Lesterhuis W.J., Barentsz J.O., Verdijk P., van Krieken J.H., Boerman O.C., Oyen W.J., Bonenkamp J.J., Boezeman J.B., Adema G.J. (2005). Magnetic resonance tracking of dendritic cells in melanoma patients for monitoring of cellular therapy. Nat. Biotechnol.

[12] Thelen M., Stein J.V. (2008). How chemokines invite leukocytes to dance. Nat. Immunol.

[13] Szatmari I., Nagy L. (2008). Nuclear receptor signalling in dendritic cells connects lipids, the genome and immune function. EMBO J.

[14] Thurnher M. (2007). Lipids in dendritic cell biology: Messengers, effectors, and antigens. J. Leukoc. Biol.

[15] Martino A. (2007). Sphingosine 1-phosphate as a novel immune regulator of dendritic cells. J. Biosci.

[16] Szatmari I., Rajnavolgyi E., Nagy L. (2006). PPARgamma, a lipid-activated transcription factor as a regulator of dendritic cell function. Ann. N. Y. Acad. Sci.

[17] Gualde N., Harizi H. (2004). Prostanoids and their receptors that modulate dendritic cell-mediated immunity. Immunol. Cell Biol.

[18] Singh P., Hoggatt J., Hu P., Speth J.M., Fukuda S., Breyer R.M., Pelus L.M. (2012). Blockade of prostaglandin E2 signaling through EP1 and EP3 receptors attenuates Flt3L-dependent dendritic cell development from hematopoietic progenitor cells. Blood.

[19] Legler D.F., Krause P., Scandella E., Singer E., Groettrup M. (2006). Prostaglandin E2 is generally required for human dendritic cell migration and exerts its effect via EP2 and EP4 receptors. J. Immunol.

[20] Van Helden S.F., Krooshoop D.J., Broers K.C., Raymakers R.A., Figdor C.G., van Leeuwen F.N. (2006). A critical role for prostaglandin E2 in podosome dissolution and induction of high-speed migration during dendritic cell maturation. J. Immunol.

[21] Van Helden S.F., Oud M.M., Joosten B., Peterse N., Figdor C.G., van Leeuwen F.N. (2008). PGE2-mediated podosome loss in dendritic cells is dependent on actomyosin contraction downstream of the RhoA-Rho-kinase axis. J. Cell Sci.

[22] Khayrullina T., Yen J.H., Jing H., Ganea D. (2008). *In vitro* differentiation of dendritic cells in the presence of prostaglandin E2 alters the IL-12/IL-23 balance and promotes differentiation of Th17 cells. J. Immunol.

[23] Harizi H., Juzan M., Grosset C., Rashedi M., Gualde N. (2001). Dendritic cells issued *in vitro* from bone marrow produce PGE(2) that contributes to the immunomodulation induced by antigen-presenting cells. Cell Immunol.

[24] Boullart A.C., Aarntzen E.H., Verdijk P., Jacobs J.F., Schuurhuis D.H., Benitez-Ribas D., Schreibelt G., van de Rakt M.W., Scharenborg N.M., de Boer A. (2008). Maturation of monocyte-derived dendritic cells with Toll-like receptor 3 and 7/8 ligands combined with prostaglandin E2 results in high interleukin-12 production and cell migration. Cancer Immunol. Immunother.

[25] Kalinski P., Hilkens C.M., Snijders A., Snijdewint F.G., Kapsenberg M.L. (1997). Dendritic cells, obtained from peripheral blood precursors in the presence of PGE2, promote Th2 responses. Adv. Exp. Med. Biol.

[26] Coleman R.A., Smith W.L., Narumiya S. (1994). International Union of Pharmacology classification of prostanoid receptors: Properties, distribution, and structure of the receptors and their subtypes. Pharmacol. Rev.

[27] Smith W.L. (1992). Prostanoid biosynthesis and mechanisms of action. Am. J. Physiol.

[28] Samuelsson B., Morgenstern R., Jakobsson P.J. (2007). Membrane prostaglandin E synthase-1: A novel therapeutic target. Pharmacol. Rev.

[29] Tai H.H., Ensor C.M., Tong M., Zhou H., Yan F (2002). Prostaglandin catabolizing enzymes. Prostaglandins Other Lipid Mediat..

[30] Tai H.H., Cho H., Tong M., Ding Y. (2006). NAD+-linked 15-hydroxyprostaglandin dehydrogenase: Structure and biological functions. Curr. Pharm. Des.

[31] Bhattacharya M., Peri K., Ribeiro-da-Silva A., Almazan G., Shichi H., Hou X., Varma D.R., Chemtob S. (1999). Localization of functional prostaglandin E2 receptors EP3 and EP4 in the nuclear envelope. J. Biol. Chem.

[32] Legler D.F., Bruckner M., Uetz-von A.E., Krause P. (2009). Prostaglandin E2 at new glance: Novel insights in functional diversity offer therapeutic chances. Int. J. Biochem. Cell Biol.

[33] Fujino H., Regan J.W. (2006). EP(4) prostanoid receptor coupling to a pertussis toxin-sensitive inhibitory G protein. Mol. Pharmacol.

[34] Matsuoka Y., Furuyashiki T., Bito H., Ushikubi F., Tanaka Y., Kobayashi T., Muro S., Satoh N., Kayahara T., Higashi M. (2003). Impaired adrenocorticotropic hormone response to bacterial endotoxin in mice deficient in prostaglandin E receptor EP1 and EP3 subtypes. Proc. Natl. Acad. Sci. USA.

[35] Matsuoka Y., Furuyashiki T., Yamada K., Nagai T., Bito H., Tanaka Y., Kitaoka S., Ushikubi F., Nabeshima T., Narumiya S. (2005). Prostaglandin E receptor EP1 controls impulsive behavior under stress. Proc. Natl. Acad. Sci. USA.

[36] Hizaki H., Segi E., Sugimoto Y., Hirose M., Saji T., Ushikubi F., Matsuoka T., Noda Y., Tanaka T., Yoshida N. (1999). Abortive expansion of the cumulus and impaired fertility in mice lacking the prostaglandin E receptor subtype EP2. Proc. Natl. Acad. Sci.

[37] Takeuchi K., Ukawa H., Kato S., Furukawa O., Araki H., Sugimoto Y., Ichikawa A., Ushikubi F., Narumiya S. (1999). Impaired duodenal bicarbonate secretion and mucosal integrity in mice lacking prostaglandin E, Äìreceptor subtype EP3. Gastroenterology.

[38] Yoshida K., Oida H., Kobayashi T., Maruyama T., Tanaka M., Katayama T., Yamaguchi K., Segi E., Tsuboyama T., Matsushita M. (2002). Stimulation of bone formation and prevention of bone loss by prostaglandin E EP4 receptor activation. Proc. Natl. Acad. Sci. USA.

[39] Kalinski P. (2012). Regulation of immune responses by prostaglandin E2. J. Immunol.

[40] Sreeramkumar V., Fresno M., Cuesta N. (2012). Prostaglandin E2 and T cells: Friends or foes?. Immunol. Cell Biol.

[41] Yao C., Sakata D., Esaki Y., Li Y., Matsuoka T., Kuroiwa K., Sugimoto Y., Narumiya S. (2009). Prostaglandin E2-EP4 signaling promotes immune inflammation through Th1 cell differentiation and Th17 cell expansion. Nat. Med.

[42] Boniface K., Bak-Jensen K.S., Li Y., Blumenschein W.M., McGeachy M.J., McClanahan T.K., McKenzie B.S., Kastelein R.A., Cua D.J. (2009). Prostaglandin E2 regulates Th17 cell differentiation and function through cyclic AMP and EP2/EP4 receptor signaling. J. Exp. Med.

[43] Kabashima K., Sakata D., Nagamachi M., Miyachi Y., Inaba K., Narumiya S. (2003). Prostaglandin E2-EP4 signaling initiates skin immune responses by promoting migration and maturation of Langerhans cells. Nat. Med.

[44] Narumiya S. (2003). Prostanoids in immunity: Roles revealed by mice deficient in their receptors. Life Sci.

[45] Honda A., Sugimoto Y., Namba T., Watabe A., Irie A., Negishi M., Narumiya S., Ichikawa A. (1993). Cloning and expression of a cDNA for mouse prostaglandin E receptor EP2 subtype. J. Biol. Chem.

[46] Regan J.W., Bailey T.J., Pepperl D.J., Pierce K.L., Bogardus A.M., Donello J.E., Fairbairn C.E., Kedzie K.M., Woodward D.F., Gil D.W. (1994). Cloning of a novel human prostaglandin receptor with characteristics of the pharmacologically defined EP2 subtype. Mol. Pharmacol.

[47] Fujino H., Xu W., Regan J.W. (2003). Prostaglandin E2 induced functional expression of early growth response factor-1 by EP4, but not EP2, prostanoid receptors via the phosphatidylinositol 3-kinase and extracellular signal-regulated kinases. J. Biol. Chem.

[48] Fujino H., West K.A., Regan J.W. (2002). Phosphorylation of glycogen synthase kinase-3 and stimulation of T-cell factor signaling following activation of EP2 and EP4 prostanoid receptors by prostaglandin E-2. J. Biol. Chem.

[49] Leduc M., Breton B., Gales C., le Gouill C., Bouvier M., Chemtob S., Heveker N. (2009). Functional selectivity of natural and synthetic prostaglandin EP4 receptor ligands. J. Pharmacol. Exp. Ther.

[50] Fujino H., Salvi S., Regan J.W. (2005). Differential regulation of phosphorylation of the cAMP response element-binding protein after activation of EP2 and EP4 prostanoid receptors by prostaglandin E2. Mol. Pharmacol.

[51] Naraba H., Yokoyama C., Tago N., Murakami M., Kudo I., Fueki M., Oh-Ishi S., Tanabe T. (2002). Transcriptional regulation of the membrane-associated prostaglandin E2 synthase gene. Essential role of the transcription factor Egr-1. J. Biol. Chem.

[52] Desai S., Ashby B. (2001). Agonist-Induced internalization and mitogen-activated protein kinase activation of the human prostaglandin EP4 receptor. FEBS Lett.

[53] Neuschafer-Rube F., Oppermann M., Moller U., Boer U., Puschel G.P. (1999). Agonist-Induced phosphorylation by G protein-coupled receptor kinases of the EP4 receptor carboxyl-terminal domain in an EP3/EP4 prostaglandin E(2) receptor hybrid. Mol. Pharmacol.

[54] Penn R.B., Pascual R.M., Kim Y.M., Mundell S.J., Krymskaya V.P., Panettieri R.A., Benovic J.L. (2001). Arrestin specificity for G protein-coupled receptors in human airway smooth muscle. J. Biol. Chem..

[55] Desai S., April H., Nwaneshiudu C., Ashby B. (2000). Comparison of agonist-induced internalization of the human EP2 and EP4 prostaglandin receptors: Role of the carboxyl terminus in EP4 receptor sequestration. Mol. Pharmacol.

[56] North T.E., Goessling W., Walkley C.R., Lengerke C., Kopani K.R., Lord A.M., Weber G.J., Bowman T.V., Jang I.-H., Grosser T. (2007). Prostaglandin E2 regulates vertebrate haematopoietic stem cell homeostasis. Nature.

[57] Lord A.M., North T.E., Zon L.I. (2007). Prostaglandin E2: Making more of your marrow. Cell Cycle.

[58] Goessling W., Allen R.S., Guan X., Jin P., Uchida N., Dovey M., Harris J.M., Metzger M.E., Bonifacino A.C., Stroncek D. (2011). Prostaglandin E2 enhances human cord blood stem cell xenotransplants and shows long-term safety in preclinical nonhuman primate transplant models. Cell Stem Cell.

[59] Hoggatt J., Singh P., Sampath J., Pelus L.M. (2009). Prostaglandin E2 enhances hematopoietic stem cell homing, survival, and proliferation. Blood.

[60] Ikushima Y.M., Arai F., Hosokawa K., Toyama H., Takubo K., Furuyashiki T., Narumiya S., Suda T. (2013). Prostaglandin E2 regulates murine hematopoietic stem/progenitor cells directly via EP4 receptor and indirectly through mesenchymal progenitor cells. Blood.

[61] Porter R.L., Georger M., Bromberg O., McGrath K.E., Frisch B.J., Becker M.W., Calvi L.M. (2013). Prostaglandin E2 increases hematopoietic stem cell survival and accelerates hematopoietic recovery after radiation injury. Stem Cells.

[62] Frisch B.J., Porter R.L., Gigliotti B.J., Olm-Shipman A.J., Weber J.M., O’Keefe R.J., Jordan C.T., Calvi L.M. (2009). *In vivo* prostaglandin E2 treatment alters the bone marrow microenvironment and preferentially expands short-term hematopoietic stem cells. Blood.

[63] Goessling W., North T.E., Loewer S., Lord A.M., Lee S., Stoick-Cooper C.L., Weidinger G., Puder M., Daley G.Q., Moon R.T. (2009). Genetic interaction of PGE2 and Wnt signaling regulates developmental specification of stem cells and regeneration. Cell.

[64] Al-Kharusi M.R., Smartt H.J., Greenhough A., Collard T.J., Emery E.D., Williams A.C., Paraskeva C (2013). LGR5 promotes survival in human colorectal adenoma cells and is upregulated by PGE2: Implications for targeting adenoma stem cells with NSAIDs. Carcinogenesis.

[65] Romani N., Gruner S., Brang D., Kampgen E., Lenz A., Trockenbacher B., Konwalinka G., Fritsch P.O., Steinman R.M., Schuler G. (1994). Proliferating dendritic cell progenitors in human blood. J. Exp. Med.

[66] Romani N., Reider D., Heuer M., Ebner S., Kampgen E., Eibl B., Niederwieser D., Schuler G. (1996). Generation of mature dendritic cells from human blood. An improved method with special regard to clinical applicability. J. Immunol. Methods.

[67] Tacken P.J., de Vries I.J., Torensma R., Figdor C.G. (2007). Dendritic-Cell immunotherapy: From *ex vivo* loading to *in vivo* targeting. Nat. Rev. Immunol.

[68] Cheong C., Matos I., Choi J.H., Dandamudi D.B., Shrestha E., Longhi M.P., Jeffrey K.L., Anthony R.M., Kluger C., Nchinda G. (2010). Microbial stimulation fully differentiates monocytes to DC-SIGN/CD209(+) dendritic cells for immune T cell areas. Cell.

[69] Spaggiari G.M., Abdelrazik H., Becchetti F., Moretta L. (2009). MSCs inhibit monocyte-derived DC maturation and function by selectively interfering with the generation of immature DCs: Central role of MSC-derived prostaglandin E2. Blood.

[70] Obermajer N., Kalinski P. (2012). Key role of the positive feedback between PGE(2) and COX2 in the biology of myeloid-derived suppressor cells. Oncoimmunology.

[71] Van Helden S.F., van den Dries K., Oud M.M., Raymakers R.A., Netea M.G., van Leeuwen F.N., Figdor C.G. (2010). TLR4-Mediated podosome loss discriminates gram-negative from gram-positive bacteria in their capacity to induce dendritic cell migration and maturation. J. Immunol.

[72] Scandella E., Men Y., Gillessen S., Forster R., Groettrup M. (2002). Prostaglandin E2 is a key factor for CCR7 surface expression and migration of monocyte-derived dendritic cells. Blood.

[73] Yen J.H., Khayrullina T., Ganea D. (2008). PGE2-induced metalloproteinase-9 is essential for dendritic cell migration. Blood.

[74] Yen J.H., Kocieda V.P., Jing H., Ganea D. (2011). Prostaglandin E2 induces matrix metalloproteinase 9 expression in dendritic cells through two independent signaling pathways leading to activator protein 1 (AP-1) activation. J. Biol. Chem.

[75] Krause P., Bruckner M., Uermosi C., Singer E., Groettrup M., Legler D.F. (2009). Prostaglandin E(2) enhances T-cell proliferation by inducing the costimulatory molecules OX40L, CD70, and 4–1BBL on dendritic cells. Blood.

[76] Doyen V., Rubio M., Braun D., Nakajima T., Abe J., Saito H., Delespesse G., Sarfati M. (2003). Thrombospondin 1 is an autocrine negative regulator of human dendritic cell activation. J. Exp. Med.

[77] Krause P., Singer E., Darley P.I., Klebensberger J., Groettrup M., Legler D.F. (2007). Prostaglandin E2 is a key factor for monocyte-derived dendritic cell maturation: Enhanced T cell stimulatory capacity despite IDO. J. Leukoc. Biol.

[78] De Smedt T., van Mechelen M., de Becker G., Urbain J., Leo O., Moser M. (1997). Effect of interleukin-10 on dendritic cell maturation and function. Eur. J. Immunol.

[79] Kalinski P., Hilkens C.M., Snijders A., Snijdewint F.G., Kapsenberg M.L. (1997). IL-12-Deficient dendritic cells, generated in the presence of prostaglandin E2, promote type 2 cytokine production in maturing human naive T helper cells. J. Immunol.

[80] Kalim K.W., Groettrup M. (2013). Prostaglandin E2 inhibits IL-23 and IL-12 production by human monocytes through down-regulation of their common p40 subunit. Mol. Immunol.

[81] Poloso N.J., Urquhart P., Nicolaou A., Wang J., Woodward D.F. (2013). PGE(2) differentially regulates monocyte-derived dendritic cell cytokine responses depending on receptor usage (EP(2)/EP(4)). Mol. Immunol.

[82] Kocieda V.P., Adhikary S., Emig F., Yen J.H., Toscano M.G., Ganea D. (2012). Prostaglandin E2-induced IL-23p19 subunit is regulated by cAMP-responsive element-binding protein and C/AATT enhancer-binding protein beta in bone marrow-derived dendritic cells. J. Biol. Chem.

[83] Fabricius D., Neubauer M., Mandel B., Schutz C., Viardot A., Vollmer A., Jahrsdorfer B., Debatin K.M. (2010). Prostaglandin E2 inhibits IFN-alpha secretion and Th1 costimulation by human plasmacytoid dendritic cells via E-prostanoid 2 and E-prostanoid 4 receptor engagement. J. Immunol.

[84] Fabricius D., O’Dorisio M.S., Blackwell S., Jahrsdorfer B. (2006). Human plasmacytoid dendritic cell function: Inhibition of IFN-alpha secretion and modulation of immune phenotype by vasoactive intestinal peptide. J. Immunol.

[85] Son Y., Ito T., Ozaki Y., Tanijiri T., Yokoi T., Nakamura K., Takebayashi M., Amakawa R., Fukuhara S. (2006). Prostaglandin E2 is a negative regulator on human plasmacytoid dendritic cells. Immunology.

[86] Strong P.N., Goerke J., Oberg S.G., Kelly R.B. (1976). Beta-Bungarotoxin, a pre-synaptic toxin with enzymatic activity. Proc. Natl. Acad. Sci. USA.

